# Complementary Diagnostic Roles of Non-Invasive Prenatal Testing, Chromosomal Microarray Analysis, and Karyotyping in 14,011 High-Risk Pregnancies: A Retrospective Cohort Study with Combined Analyses

**DOI:** 10.3390/jcm15166240

**Published:** 2026-08-12

**Authors:** Seungyeon Lee, Suhng Wook Kim, Eunhee Lee, Sanggon Lee, Sunghee Han

**Affiliations:** 1 Department of Health and Safety Convergence Science, Graduate School, Korea University, Seoul 02841, Republic of Korea; leesy4205@gmail.com (S.L.);; 2 Division of Cytogenetics, Department of Laboratory Medicine, Green Cross Laboratories, 107 Ihyeon-ro 30 beon-gil, Giheung-gu, Yongin-si 16924, Republic of Korea

**Keywords:** non-invasive prenatal testing, chromosomal microarray analysis, chromosome analysis, karyotyping, amniocentesis, high-risk pregnancy, chromosomal abnormalities

## Abstract

**Background/Objectives**: Prenatal chromosomal assessment can benefit from the complementary use of non-invasive prenatal testing (NIPT), chromosomal microarray analysis (CMA), and karyotyping because each test provides different information. **Methods**: We retrospectively analyzed 14,011 fetus-specific amniotic fluid cases obtained through second-trimester amniocentesis between 2014 and 2023. The study included overlapping subgroups tested by NIPT and karyotyping (*n* = 1300), CMA and karyotyping (*n* = 444), or all three methods (*n* = 78). **Results**: Karyotyping identified fetal chromosomal abnormalities in 1288 cases (9.2%). Among 983 evaluable cases in the clinically selected NIPT–karyotyping subgroup referred for invasive diagnosis by amniocentesis, the positive predictive value was 88.8% for trisomy 21, 66.7% for trisomy 18, 21.4% for trisomy 13, and 37.0% for sex chromosome abnormalities. Because these estimates were derived from a highly selected referral cohort rather than an unselected prenatal screening population, the PPV and NPV values should be interpreted within this referral setting and should not be generalized to unselected prenatal screening populations. Low-risk or inconclusive NIPT results did not completely exclude fetal chromosomal abnormalities. CMA detected pathogenic or likely pathogenic copy-number findings in 17 of 397 cases with normal karyotypes and provided additional molecular information in selected cases with abnormal karyotypes. Conversely, karyotyping identified 11 abnormalities among 388 cases with only likely benign, benign, or normal CMA results, including 10 apparently balanced reciprocal translocations and one diploid–tetraploid mosaicism. **Conclusions**: These findings show that NIPT, CMA, and karyotyping provide complementary rather than interchangeable information. High-risk NIPT results should be confirmed by invasive diagnostic testing, while CMA and karyotyping should be selected according to the clinical indication and suspected type of abnormality.

## 1. Introduction

South Korea has experienced persistently low fertility, while the proportion of births to women of advanced maternal age has increased. Against this demographic background, accessible prenatal screening and diagnostic services have become an increasingly important clinical and policy consideration. Pregnancies referred for high-risk prenatal evaluation commonly involve advanced maternal age (AMA; ≥35 years), abnormal ultrasound findings, or positive serum screening, which are associated with an increased risk of fetal chromosomal abnormalities. According to national birth statistics [1], women aged ≥35 years accounted for 35.9% of births in South Korea in 2024. In our 10-year cohort (2014–2023), women aged ≥35 years accounted for 57.7% of cases, consistent with referral enrichment for invasive testing among women of advanced maternal age [2]. Referral pathways, however, are shaped not only by individual clinical risk but also by the organization, timing, availability, and cost of screening programs. A nationwide analysis of Poland’s publicly funded prenatal screening program showed that program coverage, reimbursement, and access to time-sensitive testing were associated with screening uptake and the use of invasive diagnostic procedures [3]. These findings suggest that program design and real-world accessibility may influence referral pathways from prenatal screening to invasive diagnosis and, consequently, access to downstream genetic testing. Despite the widespread use of karyotyping, CMA, and NIPT, comparative evidence from large karyotyping cohorts incorporating paired NIPT–karyotyping and CMA–karyotyping analyses in clinically selected subgroups remains limited. Such evidence is needed to clarify the complementary roles of these methods in real-world prenatal care.

Currently, prenatal genetic testing is conducted using three complementary methodologies, each with distinct capabilities and limitations. G-banded karyotyping, with an approximate resolution of 5–6 Mb, has long served as a standard cytogenetic method for detecting large chromosomal abnormalities, balanced translocations, and mosaicism. However, it is inadequate for detecting submicroscopic copy number variations (CNVs) and is subject to inter-laboratory variability due to the subjective interpretation of banding patterns [4]. CMA addresses this resolution limitation by detecting genome-wide CNVs with a resolution of approximately 100 kb, providing detailed information on the size, gene content, and cytogenetic location of deletions or duplications [5,6], as well as regions of homozygosity suggestive of consanguinity or uniparental disomy [7,8]. Since the discovery of cell-free fetal DNA (cfDNA) by Lo et al. [9] and the introduction of massively parallel sequencing, NIPT has emerged as a first-tier screening test for common fetal aneuploidies, although its performance varies across target conditions [10,11]. In South Korea, abnormal NIPT results have become an important indication for amniocentesis in contemporary prenatal practice [2]. However, NIPT remains vulnerable to false-positive results due to confined placental mosaicism (CPM) and has limited resolution for detecting subchromosomal CNVs or structural abnormalities [12,13,14].

Thus, the three methodologies provide distinct but complementary information. Karyotyping detects large numerical and structural chromosomal abnormalities, including balanced rearrangements and certain forms of mosaicism, but has limited resolution for submicroscopic genomic alterations. CMA enables high-resolution detection and characterization of pathogenic CNVs but cannot reliably identify balanced rearrangements or fully characterize certain mosaic and polyploid abnormalities. NIPT provides non-invasive screening for common fetal aneuploidies, although its performance varies by target condition and its results require confirmation by invasive diagnostic testing. Although these methods have been extensively evaluated individually, evidence describing their complementary performance within a large karyotyping cohort and clinically selected paired-test subgroups remains limited. Therefore, this study aimed to characterize karyotype findings in 14,011 fetus-specific amniotic fluid cases from high-risk pregnancies and to evaluate paired NIPT–karyotyping and CMA–karyotyping results in clinically selected subgroups, including a smaller subgroup that underwent all three tests. By examining condition-specific performance and concordant and discordant findings across the testing modalities, we sought to provide clinically relevant evidence for interpreting prenatal genetic test results and to clarify how NIPT, CMA, and karyotyping may be selected and integrated according to the clinical question.

## 2. Materials and Methods

### 2.1. Participants and Cohort Construction

Clinical and laboratory data were retrospectively collected at Green Cross Laboratories, a nationwide clinical reference laboratory in South Korea. The primary cohort comprised 14,011 fetus-specific cases evaluated by second-trimester amniotic fluid karyotyping between January 2014 and December 2023. Specimens were referred to Green Cross Laboratories from obstetric clinics, general hospitals, and tertiary referral centers throughout South Korea. All karyotyping, NIPT, and CMA results included in this study were generated at Green Cross Laboratories. The tests were ordered by obstetricians at the referring institutions according to the clinical circumstances of each pregnancy and the physician’s clinical judgment.

The unit of analysis was the fetus-specific amniotic fluid sample. Each separately collected amniotic fluid specimen and its corresponding fetal test result constituted one analytical case. In singleton pregnancies, one case generally represented one fetus. In multiple gestations, amniotic fluid specimens collected separately from different fetuses were entered and analyzed as separate cases; therefore, more than one analytical case could originate from the same pregnancy. When only one fetus in a multiple gestation underwent amniocentesis, only that fetal sample was included. Accordingly, the 14,011 records represent fetus-specific amniotic fluid cases rather than 14,011 unique pregnancies.

From the primary karyotyping cohort, smaller, clinically selected, and overlapping subgroups were identified through retrospective record linkage: 1300 cases with both NIPT and karyotyping, 444 cases with both CMA and karyotyping, and 78 cases with all three tests. The 78 cases were included in both paired subgroups and were also analyzed separately. The anonymized dataset was manually reviewed to distinguish repeated records for the same fetus-specific case from records arising from separately sampled fetuses in a multiple gestation or from a subsequent pregnancy. Duplicate records for the same fetus-specific case were removed, whereas separately sampled fetuses and subsequent pregnancies were retained as separate analytical cases.

Clinical indications included abnormal MSS or NIPT results, AMA, abnormal US findings, relevant reproductive or family history, IVF, maternal anxiety, maternal fragile X carrier status, recurrent miscarriage, and twin pregnancy (Table 1). When multiple indications were documented, the primary indication recorded in the referral information was used for classification. Clinical indications were additionally reviewed in the 444 cases that underwent both CMA and karyotyping.

The study protocol was approved by the Institutional Review Board of Green Cross Laboratories (GCL-2025-1033-01). The requirement for informed consent was waived because of the retrospective nature of the study and the use of anonymized data.

### 2.2. Karyotype Analysis

During the study period, the combination of culture media was changed due to supply constraints. Amniotic fluid cells were initially cultured using BIO-AMF™-2 medium (Biological Industries Ltd., Beit Haemek, Israel) and KREAvital Prenatal Medium PLUS (Complete; Cat. No. KBI-90013; Kreatech Biotechnology B.V., Amsterdam, The Netherlands). They were subsequently cultured using AmnioPrime medium (Capricorn Scientific GmbH, Ebsdorfergrund, Germany) and Amniogrow Plus medium (Cytogen GmbH, Greven, Germany). Cultures were incubated for 9–14 days or until at least 15 colonies had developed. Chromosome preparations were obtained using standard cytogenetic procedures. For each case, 15–20 metaphase cells from two independent cultures were analyzed by Giemsa–trypsin–Giemsa (GTG) banding. Karyotypes were described according to the version of the International System for Human Cytogenomic Nomenclature (ISCN) in effect at the time of testing. Abnormalities were classified as numerical or structural and as autosomal or sex-chromosome abnormalities; structural abnormalities were further categorized as balanced or unbalanced. For presentation in this study, karyotype descriptions were standardized according to ISCN 2024 [15]. Cytogenetic variants regarded as normal polymorphic variants were excluded from the analysis.

### 2.3. NIPT Analysis

The paired NIPT–karyotyping analysis included a clinically selected subgroup of 1300 cases from the primary karyotyping cohort. When more than one NIPT result was available for the same pregnancy, the latest result obtained before amniocentesis was used because repeat blood sampling was frequently requested after an initial inconclusive result, and the latest pre-amniocentesis result represented the result available for subsequent clinical decision-making.

Maternal peripheral blood (10 mL) was collected in Cell-Free DNA BCT™ tubes (Streck, Omaha, NE, USA). Blood samples were centrifuged at 1600× *g* for 10 min, followed by secondary centrifugation of the separated plasma at 3000× *g* for 10 min. cfDNA was extracted from 1 mL of plasma using the Tiangen Micro DNA Kit (Tiangen Biotech Co., Ltd., Beijing, China), and sequencing libraries were prepared using the TruSeq Nano DNA Kit (Illumina, San Diego, CA, USA). Sequencing was performed on the NextSeq 500 platform (Illumina, San Diego, CA, USA) in 75-bp single-end mode, generating approximately 12 million reads per sample. All clinical NIPT results included in the present paired cohort were generated using the NextSeq 500-based workflow described below.

Generated reads were aligned to the human reference genome (hg19) using BWA-MEM (v0.7.5) with default parameters. PCR duplicate reads were removed using Picard (v1.96), and reads with mapping quality scores below 60 were excluded using SAMtools (v1.2). GC-content and mappability biases were corrected using the readDepth package (v0.9.8.4) in R version 4.0.5 with default parameters [16]. Fetal fraction was estimated using SeqFF with default parameters. The artificial intelligence algorithm using cell-free DNA fragment distance (aiD-NIPT), implemented using TensorFlow v2.2.0 (Google LLC), and WisecondorX were used to assess chromosomal aneuploidies and copy-number variants, respectively. Results were considered inconclusive when the fetal fraction was <3.0%, cfDNA read-count variability was unusually high, or other quality-control criteria were not met. The specific reason for each inconclusive result and the number of repeat NIPT tests were not captured as structured variables and therefore could not be quantified.

### 2.4. Chromosomal Microarray Analysis

The paired CMA–karyotyping analysis included 444 clinically selected cases from the primary cohort. In some cases, CMA and karyotyping were ordered together using the same amniotic fluid specimen; in others, the referring institution requested an additional test after review of the initial result, and testing was performed after the availability of a retained specimen was confirmed.

CMA was performed to detect genome-wide CNVs, including microdeletions and duplications of ≥100 kb. Genomic DNA was extracted from amniotic fluid using the Chemagic DNA Blood200 Kit (Chemagen, Baesweiler, Germany) according to the manufacturer’s instructions. DNA concentration was measured using a Qubit fluorometer (Thermo Fisher Scientific, Waltham, MA, USA). When blood contamination was suspected, maternal blood DNA and amniotic fluid DNA were compared using short tandem repeat polymerase chain reaction to assess maternal cell contamination and confirm sample identity. CMA was performed using the Affymetrix CytoScan Optima array platform (Thermo Fisher Scientific, Waltham, MA, USA). DNA samples underwent enzymatic digestion, PCR amplification, purification, quantification, fragmentation, labeling, hybridization, staining, and scanning in accordance with the manufacturer’s protocol (CytoScan™ Optima Assay User Guide, Pub. No. 703280; Thermo Fisher Scientific, Waltham, MA, USA). Data were processed and analyzed using Chromosome Analysis Suite (ChAS) software (version 3.1.0.15; Thermo Fisher Scientific, Waltham, MA, USA). Detected CNVs were interpreted according to the technical standards and interpretation guidelines of the American College of Medical Genetics and Genomics and ClinGen and were classified as pathogenic (P), likely pathogenic (LP), variants of uncertain significance (VUS), likely benign (LB), or benign (B). CNV findings were interpreted in conjunction with the available clinical and cytogenetic findings. When available, parental studies were used to assess inheritance, and selected findings were further evaluated using supplementary molecular testing.

### 2.5. Statistical Analysis

Statistical analyses were performed using SPSS Statistics version 27.0 (IBM Corp., Armonk, NY, USA) and Microsoft Excel (Microsoft Corp., Redmond, WA, USA). Descriptive statistics were used to summarize the primary karyotyping cohort and each clinically selected subgroup separately. Continuous variables are presented as means, and categorical variables as numbers and percentages.

Inconclusive NIPT results (*n* = 316) were excluded from the primary performance analyses and evaluated separately. One case with a karyotype result reported as “no growth” was also excluded, leaving 983 cases for each condition-specific analysis. For each target condition, an interpretable NIPT result was classified as test-positive only when it was reported as high-risk for that condition. All other interpretable results, including low-risk results and results reported as high-risk for a different target condition, were classified as test-negative for the corresponding analysis. A false-negative result was defined as a target-specific abnormality identified by karyotyping in a case with a test-negative NIPT result for that condition. Inconclusive NIPT results were classified as neither test-positive nor test-negative and were therefore not counted as false negatives.

Sensitivity, specificity, positive predictive value (PPV), negative predictive value (NPV), and false-positive rate (FPR) were calculated separately for T21, T18, T13, and sex chromosome abnormalities using one-versus-rest 2 × 2 contingency tables, with karyotyping as the reference standard. For each condition-specific analysis, only the corresponding karyotype finding was classified as reference-positive, whereas all other evaluable karyotype findings were classified as reference-negative. For the sex chromosome analysis, numerical, mosaic, and structural sex chromosome abnormalities were classified as reference-positive. Two-sided 95% confidence intervals were calculated using the exact Clopper–Pearson method. Because each target condition was evaluated independently, no pooled overall performance estimate was calculated.

CMA findings were summarized according to normal and abnormal karyotype results among the 444 cases that underwent both tests. The 78 cases that underwent all three tests were evaluated descriptively for concordant and discordant findings.

## 3. Results

**Figure 1:**
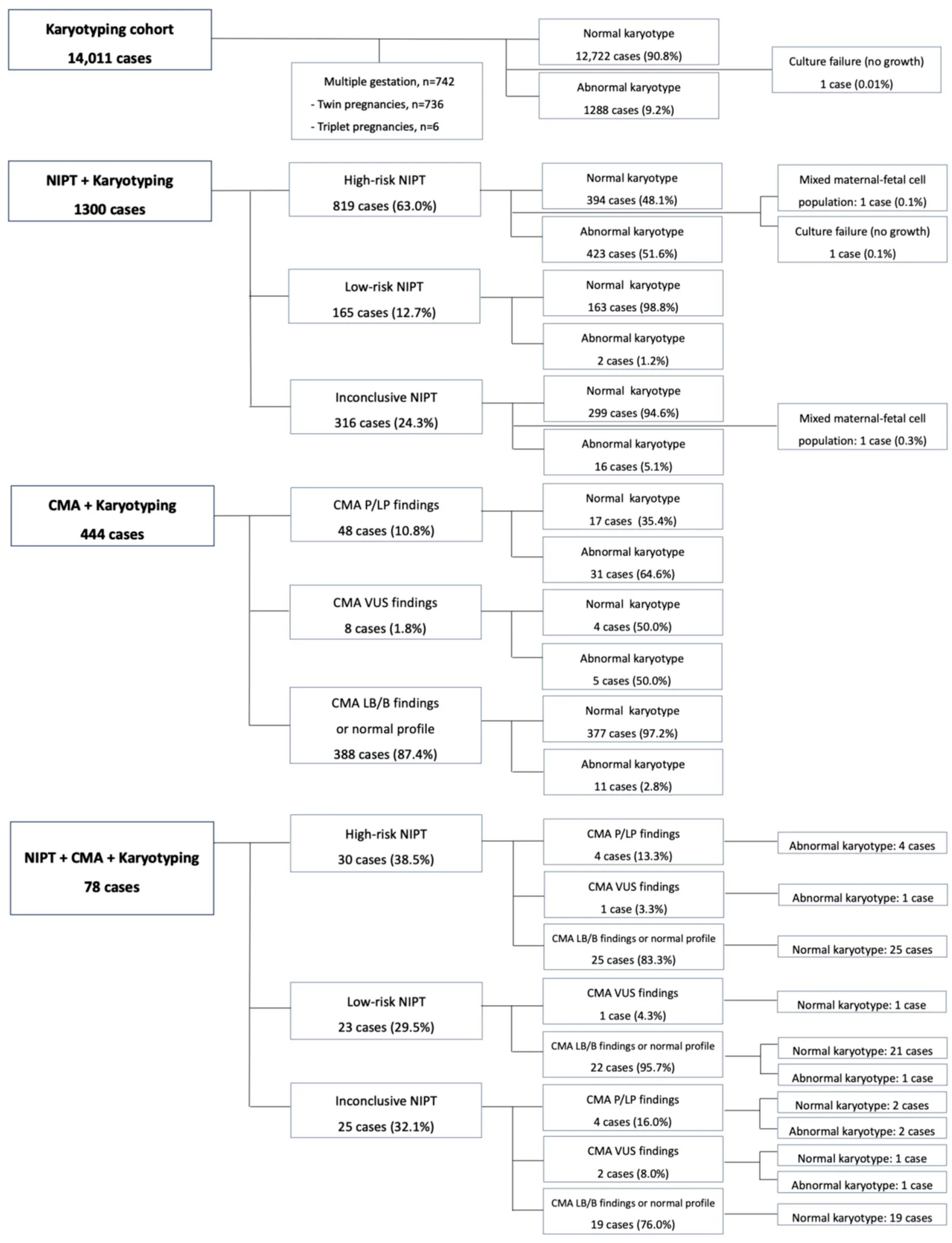
Study flow diagram of the prenatal cohort and paired genetic testing subgroups. The “Twin pregnancy” indication denotes cases for which twin gestation was recorded as the primary clinical indication; the overall cohort included 736 fetus-specific cases from twin gestations. Culture failure and mixed maternal–fetal cell population cases were presented separately from confirmed normal and abnormal fetal karyotypes. **Abbreviations:** CMA, chromosomal microarray analysis; NIPT, non-invasive prenatal testing; P, pathogenic; LP, likely pathogenic; VUS, variant of uncertain significance; LB, likely benign; B, benign.

Figure 1 summarizes the overall study population and the composition of the paired-testing subgroups. Among the 14,011 fetus-specific amniotic fluid cases included in the primary cohort and amniotic fluid karyotyping, fetal chromosomal abnormalities were identified in 1288 cases (9.2%). The dataset included 736 fetus-specific cases from twin gestations and six cases from triplet gestations. The paired NIPT–karyotyping subgroup comprised 1300 cases, including 819 high-risk, 165 low-risk, and 316 inconclusive NIPT results. The clinically selected CMA–karyotyping subgroup comprised 444 cases, of which 48 had at least one P/LP CMA finding, eight had at least one VUS, and 388 had only LB/B or normal CMA findings. 

The paired CMA–karyotyping subgroup comprised 444 cases. The distribution of primary clinical indications in this subgroup is summarized in Table 2. The most common indications were abnormal US findings (95/444, 21.4%), abnormal or nonreportable NIPT results (85/444, 19.1%), and IVF (79/444, 17.8%). Parental or family history of chromosomal abnormalities and a previous child with a chromosomal abnormality and/or intellectual disability accounted for 42 cases (9.5%) and 36 cases (8.1%), respectively. Abnormal MSS and AMA were the primary indications in 29 cases (6.5%) and 16 cases (3.6%), respectively.

Among the 14,011 cases that underwent amniotic fluid karyotyping, fetal chromosomal abnormalities were identified in 1288 cases (9.2%). Numerical abnormalities accounted for 1042 cases (80.9%), whereas structural abnormalities accounted for 246 cases (19.1%). Among the numerical abnormalities, autosomal aneuploidies were the most common, occurring in 856 cases (66.5% of all fetal chromosomal abnormalities). Trisomy 21 was the most frequent autosomal aneuploidy, identified in 627 cases (48.7%), followed by trisomy 18 in 175 (13.6%) and trisomy 13 in 28 (2.2%). Less frequent autosomal aneuploidies included trisomy 9 in 15 cases (1.2%), trisomy 22 in four (0.3%), trisomy 12 and trisomy 20 in two each (0.2% each), and trisomy 3, trisomy 7, and trisomy 15 in one each (0.1% each). Sex chromosome abnormalities were identified in 167 cases (13.0%), including Klinefelter syndrome in 67 (5.2%), triple X syndrome in 46 (3.6%), Turner syndrome in 32 (2.5%), and 47,XYY in 22 (1.7%). Polyploidy was identified in 19 cases (1.5%), comprising tetraploidy in 10 (0.8%) and triploidy in nine (0.7%).

Structural abnormalities were identified in 246 cases (19.1%), including 124 balanced rearrangements (9.6%) and 122 unbalanced rearrangements (9.5%). Balanced rearrangements comprised 58 reciprocal translocations (4.5%), 34 Robertsonian translocations (2.6%), and 32 inversions (2.5%). Among the unbalanced rearrangements, complex rearrangements were the most frequent, occurring in 30 cases (2.3%), followed by marker chromosomes in 21 (1.6%), deletions in 20 (1.6%), additional chromosomal material in 18 (1.4%), duplications in 12 (0.9%), insertions in eight (0.6%), and unbalanced translocations in six (0.5%). Dicentric chromosomes were identified in three cases (0.2%), isochromosomes in two (0.2%), and a ring chromosome and a fragile site in one case each (0.1% each) (Table 3).

Karyotype findings among the 819 cases with high-risk NIPT results and the 316 cases with inconclusive results are summarized in Table 4. Among the 285 cases with high-risk NIPT results for T21, 253 (88.8%) had target-concordant T21 findings on karyotyping, 31 (10.9%) had normal karyotypes, and one (0.4%) had a marker chromosome without T21. Among the 93 cases with high-risk results for T18, 62 (66.7%) had target-concordant T18 findings, 29 (31.2%) had normal karyotypes, and two (2.2%) had other chromosomal abnormalities. Among the 28 cases with high-risk results for T13, six (21.4%) had target-concordant T13 findings, 21 (75.0%) had normal karyotypes, and one (3.6%) had a Robertsonian translocation without trisomy 13. Among the 231 cases with high-risk NIPT results for sex chromosome abnormalities, 85 (36.8%) had sex chromosome abnormalities, 145 (62.8%) had normal karyotypes, and one (0.4%) had culture failure. After excluding the culture failure case, the PPV among 230 evaluable cases was 37.0%.

Among cases with high-risk results for microdeletions, three of 25 (12.0%) had deletions or other structural chromosomal abnormalities. Among those with high-risk results for microduplications, four of 16 (25.0%) had fetal structural chromosomal abnormalities, and one (6.3%) showed mixed maternal–fetal cell populations consistent with maternal cell contamination. Most high-risk results for rare autosomal trisomies were associated with normal karyotypes; however, target-concordant mosaic trisomies were identified for chromosomes 9, 15, 20, and 22. Among the 316 cases with inconclusive NIPT results, 299 (94.6%) had normal karyotypes, 16 (5.1%) had fetal chromosomal abnormalities, and one (0.3%) showed mixed maternal–fetal cell populations consistent with maternal cell contamination.

The condition-specific performance of NIPT for T21, T18, T13, and SCAs is presented in Table 5. Sensitivity was 99.6% for T21, 100.0% for T18, 100.0% for T13, and 95.5% for SCAs (Table 5). For T13, the estimate was based on only six karyotype-confirmed cases, with a 95% confidence interval of 54.1–100.0%. Specificity was 95.6% for T21, 96.6% for T18, 97.7% for T13, and 83.8% for SCAs. PPV varied substantially across target conditions, with values of 88.8% for T21, 66.7% for T18, 21.4% for T13, and 37.0% for SCAs. NPV ranged from 99.5% to 100.0%, whereas FPR ranged from 2.3% for T13 to 16.2% for SCAs. Because each target condition was evaluated independently using a one-versus-rest approach, no pooled performance estimate was calculated.

Of the 444 clinically selected cases evaluated by both CMA and karyotyping, 397 had normal karyotypes and 47 had abnormal karyotypes. CMA results were categorized according to the most clinically significant finding: 48 cases had at least one P/LP finding, eight had a VUS without a concurrent P/LP finding, and the remaining 388 had only LB/B findings or a normal array profile.

Pathogenic or likely pathogenic CMA findings were identified in 48 cases. Seventeen of these cases (35.4%) had normal karyotypes, whereas 31 (64.6%) had abnormal karyotypes. The abnormal karyotype findings included whole-chromosome and mosaic aneuploidies, marker chromosomes, derivative chromosomes, deletions, and additional chromosomal material. In one mosaic case, CMA detected a mosaic gain of chromosome 9 [arr(9)x2~3], whereas karyotyping identified multiple cell lines, including a trisomy 9 cell line with inv(7)(q11.23q21.2); the copy-number-neutral inversion was not identified by CMA. In a case with a marker chromosome (47,U,+mar), CMA detected a 1.8 Mb gain at 1p12p11.2 and a 1.7 Mb pathogenic loss at Xp22.31. CMA also defined the size and genomic coordinates of cytogenetically visible deletions; for example, del(15)(q11.2q13) on karyotyping corresponded to a 6.5 Mb pathogenic loss at 15q11.2q13.1. Detailed CMA findings and the corresponding karyotype results are presented in Table 6.

VUS findings were detected in eight cases, of which four (50.0%) had normal karyotypes and four (50.0%) had abnormal karyotypes. The four abnormal karyotypes comprised three marker chromosome cases, including two mosaic and one non-mosaic case, and one chromosome 2 duplication. The corresponding CMA and karyotype findings are summarized in Table 7.

Of the 388 cases with only LB/B CMA findings or a normal array profile, 11 (2.8%) had non-normal karyotypes: 10 apparently balanced reciprocal translocations and one case of diploid–tetraploid mosaicism.

Table 8 summarizes the findings from the 78 cases that underwent NIPT, CMA, and karyotyping. NIPT results were low risk in 23 cases, high risk in 30, and inconclusive in 25. In the low-risk group, 19 cases had a normal array profile and normal karyotypes, whereas three had LB or VUS CMA findings with normal karyotypes. The remaining case had a normal CMA profile [arr(1–23)x2] but diploid–tetraploid mosaicism on karyotyping [mos 92,XXXX[9]/46,XX[11]].

Of the 30 cases with high-risk NIPT results, 25 had normal CMA profiles and normal karyotypes, whereas five had abnormal karyotypes. Three of the five had concordant trisomy 21 findings across NIPT, CMA, and karyotyping. The other two comprised one marker chromosome case [46,X,+mar] and one derivative chromosome case [46,U,der(3)t(3;7)(p25;q31.3)]. In the inconclusive group, 18 cases had normal array profiles and normal karyotypes. Three had only LB or VUS CMA findings; two had normal karyotypes, whereas one had a mosaic marker chromosome on karyotyping. P/LP CMA findings were identified in the remaining four cases, two with normal karyotypes and two with abnormal karyotypes.

## 4. Discussion

This study characterized karyotype findings in 14,011 fetus-specific amniotic fluid cases from high-risk pregnancies and evaluated paired NIPT–karyotyping and CMA–karyotyping results in clinically selected subgroups, including 78 cases that underwent all three tests. The principal findings were that NIPT performance varied substantially according to the target condition and that low-risk or inconclusive NIPT results did not completely exclude fetal chromosomal abnormalities. CMA identified clinically significant genomic abnormalities that were not evident on conventional karyotyping and provided additional characterization of copy-number changes associated with abnormal karyotypes. Conversely, karyotyping identified apparently balanced rearrangements, mosaic cell populations, and polyploid abnormalities that were not detected or fully characterized by CMA. Collectively, these findings demonstrate that NIPT, CMA, and karyotyping provide distinct but complementary information and support an indication-based approach to prenatal genetic testing.

The present study demonstrated marked target-specific variation in NIPT performance across common autosomal trisomies and sex chromosome abnormalities. PPV was highest for T21 (88.8%), intermediate for T18 (66.7%), lower for SCAs (37.0%), and lowest for T13 (21.4%). The PPVs observed in our cohort were within the ranges reported in previous studies: 78.9–87.5% for T21, 25.8–75.0% for T18, 10.0–50.0% for T13, and 24.1–51.9% for SCAs [17,18,19,20,21,22,23,24,25]. Sensitivity estimates varied in statistical precision across the target conditions. The T13 estimate of 100.0% was based on only six karyotype-confirmed cases and had a wide 95% confidence interval of 54.1–100.0%. Therefore, the T13 estimate should be interpreted cautiously. Collectively, these findings indicate that NIPT performance should be evaluated separately for each target condition rather than summarized using a single overall performance estimate.

A false-negative T21 result in the present cohort further illustrates that a low-risk NIPT result does not completely exclude fetal aneuploidy. Of the 165 cases with low-risk NIPT results, two (1.2%) had abnormal karyotypes: one had trisomy 21, and the other had diploid–tetraploid mosaicism. The trisomy 21 case represented a false-negative result for the condition-specific T21 analysis, whereas diploid–tetraploid mosaicism was outside the principal target conditions evaluated by NIPT. In the condition-specific analysis, 253 true-positive results and one false-negative result were identified among 254 cases with karyotype-confirmed T21, corresponding to a sensitivity of 99.6%. Fetoplacental discordance caused by confined placental mosaicism or fetal mosaicism may contribute to discrepancies between NIPT and fetal karyotype results. Because the cfDNA analyzed by NIPT is predominantly placental in origin rather than directly derived from fetal cells, biologically driven discordance cannot be completely eliminated [13].

Inconclusive NIPT results also require careful clinical interpretation. The relatively high proportion of inconclusive results within the paired NIPT–karyotyping subgroup (316/1300, 24.3%) should not be interpreted as the overall NIPT inconclusive rate during the study period. This subgroup included only cases with both NIPT and amniotic fluid karyotyping, and inconclusive NIPT results in this referral setting frequently led to subsequent invasive diagnostic testing. Consequently, inconclusive results were disproportionately represented in the paired subgroup. The specific causes of the inconclusive results and the number of repeat NIPT tests could not be quantified because these variables had not been extracted as structured variables for the present analytic dataset. Because the latest pre-amniocentesis NIPT result was used, cases in which an initially inconclusive result became reportable after repeat blood sampling were classified according to the later reportable result. Therefore, this approach may have underestimated the frequency of initially inconclusive NIPT results within the paired subgroup. However, the magnitude of this potential underestimation could not be quantified for the same reason. Accordingly, both the proportion of inconclusive results and the observed frequency of chromosomal abnormalities among these cases should not be generalized to all pregnancies undergoing NIPT. Nevertheless, an inconclusive result should not be regarded as negative or reassuring. Its clinical significance and subsequent management should be assessed individually according to the reason for test failure, fetal fraction, gestational age, ultrasound findings, maternal BMI, the clinical indication for testing, and other relevant maternal or pregnancy-related factors. Current guidelines recommend genetic counseling, comprehensive ultrasound evaluation, and consideration of invasive diagnostic testing after a nonreportable or no-call result; repeat NIPT or an alternative screening approach may also be considered in selected clinical circumstances. Taken together, these findings reaffirm that NIPT should be interpreted as a screening rather than a diagnostic test. High-risk, low-risk, and inconclusive results should be interpreted in conjunction with target-specific performance, ultrasound findings, and the broader clinical context [26]. This broader context may also include complementary information derived from first-trimester biochemical and ultrasonographic screening parameters. In a cohort of 1164 pregnancies, lower first-trimester PAPP-A and free β-hCG concentrations and a higher ductus venosus pulsatility index were associated with selected adverse neonatal outcomes, although the prognostic discrimination of these parameters was limited, with all areas under the curve below 0.70 [27]. These observations support a multidimensional interpretation of prenatal screening but are distinct from evidence regarding the condition-specific performance of NIPT or the diagnostic capabilities of CMA and karyotyping.

The distribution of primary indications in the CMA–karyotyping subgroup is presented descriptively in Table 2. However, because the subgroup was clinically selected, several cases had multiple indications, and only the primary referral indication was retained for classification; indication-stratified P/LP proportions would not represent indication-specific CMA diagnostic yields. The incremental diagnostic contribution of CMA differed according to the paired karyotype result in this clinically selected subgroup. Among the 397 cases with normal karyotypes, 17 (4.3%) had at least one P/LP CMA finding, demonstrating that CMA identified clinically significant genomic abnormalities not detectable by conventional karyotyping. This finding is consistent with previous paired prenatal studies showing additional clinically relevant CMA findings in fetuses with normal karyotypes [28]. However, direct numerical comparison is limited by differences in case selection and the classification of CMA findings, and the 4.3% observed in the present subgroup should not be interpreted as a population-level incremental diagnostic yield. Among the 47 cases with abnormal karyotypes, 31 (66.0%) had at least one P/LP CMA finding. In this setting, CMA contributed principally by defining the genomic coordinates and estimated sizes of copy-number changes associated with cytogenetically visible or structurally complex abnormalities. For example, CMA detected a 1.8 Mb gain at 1p12p11.2 and a 1.7 Mb pathogenic loss at Xp22.31 in a case with a marker chromosome [47,U,+mar]. In another case, a cytogenetically visible deletion [46,U,del(15)(q11.2q13)] corresponded to a 6.5 Mb pathogenic loss at 15q11.2q13.1 on CMA. These findings demonstrate two distinct contributions of CMA: the detection of clinically significant genomic abnormalities in cases with normal karyotypes and the molecular characterization of copy-number changes in cases with abnormal karyotypes.

VUS findings further illustrate the importance of integrating CMA results with cytogenetic and clinical information. A VUS without a concurrent P/LP finding was identified in eight of the 444 cases (1.8%). Four of these cases had normal karyotypes, whereas four had abnormal karyotypes. The four abnormal karyotypes comprised three marker chromosome cases, including two mosaic and one non-mosaic case, and one case of chromosome 2 duplication. Furthermore, a CMA finding should not be assumed to correspond to a cytogenetic abnormality unless the genomic relationship between the two findings has been clearly demonstrated. VUS findings should therefore be interpreted cautiously in conjunction with fetal ultrasound findings, parental studies and inheritance status, when available, and the broader clinical context.

Despite its higher resolution for copy-number abnormalities, CMA did not detect or fully characterize all chromosomal abnormalities identified by karyotyping. Of the 388 cases with only LB/B CMA findings or a normal array profile, 11 (2.8%) had non-normal karyotypes: 10 apparently balanced reciprocal translocations and one case of diploid–tetraploid mosaicism. Previous paired analyses have similarly shown that balanced translocations, inversions, complex karyotypes, and chimeric cell populations may not be accurately resolved by CMA [28]. In the present cohort, the diploid–tetraploid mosaicism was detected by karyotyping despite a normal CMA profile [arr(1–23)x2], further illustrating the value of karyotyping for assessing mosaic and polyploid abnormalities. The clinically selected subgroup of 78 cases that underwent all three tests further illustrated both concordance and discordance across the three testing modalities (Table 8). Three cases showed concordant T21 findings across NIPT, CMA, and karyotyping. In contrast, the diploid–tetraploid mosaicism case had a low-risk NIPT result and a normal CMA profile, whereas another low-risk case had a normal karyotype but a copy-neutral region of homozygosity detected by CMA. The remaining two of the five cases with high-risk NIPT results and abnormal karyotypes involved a marker chromosome and a derivative chromosome, for which CMA provided additional copy-number information. These findings reflect the distinct biological substrates and analytical scopes of the three methods: NIPT primarily assesses placental cfDNA, CMA detects genome-wide copy-number changes and selected copy-neutral findings at high resolution, and karyotyping evaluates chromosome number, structural architecture, and cell-line composition, including mosaic and polyploid abnormalities. Accordingly, discordant findings should be interpreted in relation to the capabilities and limitations of each test and the broader clinical context rather than being automatically attributed to laboratory error [29,30,31].

This study has several strengths, including the large, practice-based cohort comprising 14,011 fetus-specific amniotic fluid cases and the detailed paired analyses of NIPT with karyotyping and CMA with karyotyping. However, several limitations should be considered. First, the retrospective study period spanning 2014–2023 may have introduced differences over time in clinical indications and referral patterns. Second, all testing was performed at a single clinical reference laboratory, which provided analytical consistency but may limit generalizability to other laboratories and prenatal populations. Third, this was not a complete head-to-head comparison of all three modalities in the entire cohort. The paired analyses involved clinically selected and overlapping subgroups, and only 78 cases underwent all three tests. Accordingly, the NIPT performance estimates and karyotype-stratified CMA findings may have been affected by selection bias and should not be generalized to unselected prenatal populations.

An important limitation is the unusually high proportion of inconclusive NIPT results in the paired NIPT–karyotyping subgroup (316/1300, 24.3%). This proportion does not represent the laboratory-wide NIPT failure rate but reflects the retrospective construction of a subgroup restricted to cases with both NIPT and amniotic fluid karyotyping. In this referral setting, cases with inconclusive NIPT results were more likely to proceed to invasive diagnostic testing and were therefore disproportionately enriched in the paired subgroup, inflating the observed inconclusive proportion relative to an unselected NIPT population. Although this enrichment limits population-level interpretation of the inconclusive proportion, it also provided a relatively large diagnostic follow-up cohort in which fetal karyotype findings after inconclusive NIPT could be descriptively characterized. Conversely, because the latest available NIPT result before amniocentesis was used, cases with an initially inconclusive result that became reportable after repeat blood sampling were classified according to the later result. This approach may have underestimated the frequency of inconclusive results at the initial NIPT attempt within the paired subgroup. Because complete repeat-testing histories were not extracted as structured variables, the number of repeated tests and the magnitude of this potential underestimation could not be quantified. These opposing sources of selection should be considered when interpreting both the inconclusive proportion and the NIPT performance estimates in this study. In addition, the sensitivity estimate for T13 was based on only six karyotype-confirmed cases, resulting in a wide confidence interval and limited statistical precision; therefore, this estimate should be interpreted cautiously. Finally, placental studies, parental testing, investigations for uniparental disomy, and long-term postnatal follow-up were not systematically available, limiting the interpretation of some discordant, VUS, and CMA-only findings.

Despite these limitations, the findings support an indication-based and complementary approach to prenatal genetic testing. NIPT should be interpreted as a screening test, and high-risk results should be followed by genetic counseling and confirmation using an invasive diagnostic procedure before irreversible clinical decisions are made. Diagnostic testing should also be considered when clinically significant ultrasound abnormalities or other clinical findings suggest an increased risk of fetal chromosomal abnormalities, regardless of a low-risk NIPT result. Inconclusive NIPT results should not be interpreted as negative or reassuring but should prompt individualized assessment based on the reason for test failure, fetal fraction, gestational age, ultrasound findings, and relevant maternal or pregnancy-related factors. These implications are broadly consistent with current guidance from the Society for Maternal-Fetal Medicine, endorsed by the American College of Obstetricians and Gynecologists [32], and the position statement of the International Society for Prenatal Diagnosis [26]. These documents emphasize informed patient choice, appropriate genetic counseling, and confirmatory diagnostic testing following a high-chance screening result. Beyond NIPT, CMA may provide additional clinically significant genomic information by detecting submicroscopic copy-number variants that are not identifiable by conventional karyotyping, whereas karyotyping remains important for evaluating balanced rearrangements, chromosomal architecture, mosaic cell populations, and polyploid abnormalities [33]. Accordingly, the selection and integration of these methods should be guided by the specific clinical indication and the type of chromosomal abnormality under consideration rather than by the assumption that one modality can replace the others. Future multicenter prospective studies using standardized clinical and laboratory workflows, systematic placental and parental investigations, and long-term postnatal follow-up are needed to quantify the incremental contribution of each testing modality, clarify the biological mechanisms underlying discordant findings, and improve evidence-based prenatal counseling.

## 5. Conclusions

This study shows that NIPT, CMA, and karyotyping provide different but complementary information in prenatal chromosomal assessment. NIPT performance varied according to the target condition, and neither low-risk nor inconclusive results completely ruled out fetal chromosomal abnormalities. CMA identified clinically significant submicroscopic genomic changes and helped characterize selected copy-number abnormalities associated with abnormal karyotypes. In contrast, karyotyping detected apparently balanced rearrangements, mosaic cell populations, and polyploid abnormalities that CMA could miss or could not fully characterize. No single test identified the full range of prenatal chromosomal abnormalities. Therefore, high-risk NIPT results should be confirmed by invasive diagnostic testing, and CMA and karyotyping should be selected according to the clinical indication, ultrasound findings, and suspected type of abnormality. The most useful approach is to combine the appropriate methods to answer the specific diagnostic question in each pregnancy.

## Figures and Tables

**Table 1:** Characteristics of the 14,011 fetus-specific cases undergoing amniotic fluid karyotyping.

Characteristics	Value
**Total**	14,011 (100.0)
**Maternal age, years**	
Mean	36.1
<35	5931 (42.3)
≥35	8080 (57.7)
**Gestational age, weeks**	
Mean	16.8
≤15 weeks	573 (4.1)
16 weeks	3588 (25.6)
17 weeks	5195 (37.1)
18 weeks	2682 (19.1)
19 weeks	992 (7.1)
20 weeks	361 (2.6)
≥21 weeks	620 (4.4)
**Primary c** **linical indication**	
Abnormal MSS	6516 (46.5)
AMA (≥35 years)	2649 (18.9)
Abnormal US findings	1732 (12.4)
Abnormal NIPT results	1070 (7.6)
Previous child with a chromosomal abnormality and/or intellectual disability	584 (4.2)
IVF	434 (3.1)
Parental or family history of chromosomal abnormalities	272 (1.9)
Maternal anxiety	259 (1.8)
Maternal fragile X carrier status	167 (1.2)
Recurrent miscarriage	166 (1.2)
Twin pregnancy	162 (1.2)

Data are presented as mean or *n* (%). Gestational age was determined on the date of amniocentesis and categorized by completed gestational weeks; for example, pregnancies at 16 weeks and 0–6 days were classified as 16 weeks. When multiple clinical indications were documented, the primary indication recorded in the referral information was used for classification. **Abbreviations:** AMA, advanced maternal age; IVF, in vitro fertilization; MSS, maternal serum screening; NIPT, non-invasive prenatal testing; US, ultrasound.

**Table 2:** Primary clinical indications among the 444 cases evaluated by both CMA and karyotyping.

Primary Clinical Indication	*n* (%)
Abnormal MSS	29 (6.5)
AMA (≥35 years)	16 (3.6)
Abnormal US findings	95 (21.4)
Abnormal or nonreportable NIPT results	85 (19.1)
Previous child with a chromosomal abnormality and/or intellectual disability	36 (8.1)
IVF	79 (17.8)
Parental or family history of chromosomal abnormalities	42 (9.5)
Maternal anxiety	5 (1.1)
Maternal fragile X carrier status	8 (1.8)
Recurrent miscarriage	27 (6.1)
Twin pregnancy	22 (5.0)
Total	444 (100.0)

Data are presented as *n* (%). Abnormal or nonreportable NIPT results included high-risk results, low fetal fraction, and inconclusive results. Abnormal US findings included increased nuchal translucency and fetal structural abnormalities, particularly cardiac abnormalities. The IVF category included pregnancies following the transfer of mosaic embryos. **Abbreviations:** AMA, advanced maternal age; CMA, chromosomal microarray analysis; IVF, in vitro fertilization; MSS, maternal serum screening; NIPT, non-invasive prenatal testing; US, ultrasound.

**Table 3:** Types and frequencies of fetal chromosomal abnormalities identified by karyotyping.

Karyotype Finding	*n*	%
**Numerical abnormalities**	1042	80.9
**Autosomal aneuploidies**	856	66.5
Trisomy 21	627	48.7
Trisomy 18	175	13.6
Trisomy 13	28	2.2
Trisomy 9	15	1.2
Trisomy 22	4	0.3
Trisomy 12	2	0.2
Trisomy 20	2	0.2
Trisomy 3	1	0.1
Trisomy 7	1	0.1
Trisomy 15	1	0.1
**Sex chromosome abnormalities**	167	13.0
Klinefelter syndrome	67	5.2
Triple X syndrome	46	3.6
Turner syndrome	32	2.5
47,XYY	22	1.7
**Polyploidies**	19	1.5
4n	10	0.8
3n	9	0.7
**Structural abnormalities**	246	19.1
**Balanced rearrangements**	124	9.6
Reciprocal translocation	58	4.5
Robertsonian translocation	34	2.6
Inversion	32	2.5
**Unbalanced rearrangements**	122	9.5
Marker chromosome	21	1.6
Deletion	20	1.6
Duplication	12	0.9
Addition	18	1.4
Complex rearrangement	30	2.3
Dicentric chromosome	3	0.2
Isochromosome	2	0.2
Ring chromosome	1	0.1
Insertion	8	0.6
Unbalanced translocation	6	0.5
Fragile	1	0.1
Total	1288	100.0

**Table 4:** Distribution of karyotype findings among 819 high-risk and 316 inconclusive NIPT results.

NIPT Results	NIPT Cases, *n* (%)	Karyotype Findings	Karyotype Cases, *n* (%)
**High-risk**	**819 (100.0)**		
T21	285 (34.8)	46,U	31 (10.9)
		47,U,+21	251 (88.1)
		47,U,+mar	1 (0.4)
		46,U,der(14;21)(q10;q10),+21	1 (0.4)
		47,U,+21[2]/46,U[25]	1 (0.4)
T18	93 (11.4)	46,U	29 (31.2)
		47,U,+18	59 (63.4)
		48,U,+18,+mar	1 (1.1)
		48,XXX,+18	1 (1.1)
		mos 45,X[10]/47,XX,+18[5]	1 (1.1)
		mos 45,X[5]/46,XY[15]	1 (1.1)
		69,XXY	1 (1.1)
T13	28 (3.4)	46,U	21 (75.0)
		47,U,+13	5 (17.9)
		46,U,+13,der(13;14)(q10;q10)	1 (3.6)
		45,U,der(13;22)(q10;q10)	1 (3.6)
SCA	231 (28.2)	46,U	145 (62.8)
		47,XXY	43 (18.6)
		47,XXX	21 (9.1)
		45,X	6 (2.6)
		47,XYY	6 (2.6)
		46,X,+mar	1 (0.4)
		46,X,del(X)(p10)	1 (0.4)
		mos 45,X[11]/46,X,i(X)(q10)[4]	1 (0.4)
		mos 45,X[1]/46,XX[26]	1 (0.4)
		mos 45,X[6]/46,XX[9]	1 (0.4)
		mos 45,X[3]/46,XX[12]	1 (0.4)
		mos 46,X,+mar[14]/45,X[9]	1 (0.4)
		mos 47,XXY[11]/46,XY[9]	1 (0.4)
		mos 47,XXX[22]/45,X[8]	1 (0.4)
		Culture failure (no growth)	1 (0.4)
Microdeletion	25 (3.1)	46,U	22 (88.0)
		46,U,del(11)(q24.1)	1 (4.0)
		46,U,del(15)(q11.2q13)	1 (4.0)
		46,U,add(14)(q24.3)	1 (4.0)
Microduplication	16 (2.0)	46,U	11 (68.8)
		46,U,del(4)(p16.1)	1 (6.3)
		46,U,der(3)t(3;7)(p25;q31.3)	1 (6.3)
		46,U,der(10)t(8;10)(q22.3;q25.2)	1 (6.3)
		46,U,dup(18)(q22q22)	1 (6.3)
		46,XY[17]/46,XX[3]	1 (6.3)
T2	3 (0.4)	46,U	3 (100.0)
T3	9 (1.1)	46,U	9 (100.0)
T4	1 (0.1)	46,U	1 (100.0)
T7	44 (5.4)	46,U	44 (100.0)
T8	18 (2.2)	46,U	18 (100.0)
T9	6 (0.7)	46,U	5 (83.3)
		mos 47,U,+9[10]/46,U[10]	1 (16.7)
T10	7 (0.9)	46,U	7 (100.0)
T11	1 (0.1)	46,U	1 (100.0)
T12	3 (0.4)	46,U	3 (100.0)
T14	8 (1.0)	46,U	8 (100.0)
T15	8 (1.0)	46,U	6 (75.0)
		mos 47,U,+15[4]/46,U[16]	1 (12.5)
		47,U,+mar	1 (12.5)
T16	10 (1.2)	46,U	9 (90.0)
		47,XYY	1 (10.0)
T17	1 (0.1)	46,U	1 (100.0)
T20	9 (1.1)	46,U	8 (88.9)
		mos 47,U,+20[6]/46,U[9]	1 (11.1)
T22	13 (1.6)	46,U	12 (92.3)
		mos 47,U,+22[2]/46,U[13]	1 (7.7)
**Inconclusive**	**316 (100.00)**	46,U	299 (94.6)
		46,U,add(18)(q23)	1 (0.3)
		45,U,der(13;14)(q10;q10)	1 (0.3)
		46,U,t(2;7)(p15;p13)	1 (0.3)
		46,U,t(9;17)(q13;p11.2)	1 (0.3)
		47,U,+18	1 (0.3)
		47,U,+21	1 (0.3)
		47,XXX	1 (0.3)
		47,XXY	1 (0.3)
		46,XY[53]/46,XX[4]	1 (0.3)
		mos 45,X[6]/46,XY[9]	1 (0.3)
		mos 45,X[5]/46,XX[10]	1 (0.3)
		mos 46,X,+mar[5]/46,XY[15]	1 (0.3)
		mos 46,X,+mar[10]/45,X[7]	1 (0.3)
		mos 46,U,t(4;13)(p15.2;q14.1)[1]/46,U[20]	1 (0.3)
		mos 46,U,inv(9)(p23q21.1)[9]/46,U[11]	1 (0.3)
		mos 47,XXX[10]/45,X[5]	1 (0.3)
		mos 92,XXYY[5]/46,XY[15]	1 (0.3)
Total			1135 (100.0)

Karyotype descriptions presented in this table were standardized according to ISCN 2024. U denotes an undisclosed sex chromosome complement. **Abbreviations:** T, Trisomy; SCAs, sex chromosome abnormalities.

**Table 5:** Performance of NIPT for T21, T18, T13, and SCAs in the clinically selected subgroup referred for amniocentesis.

Target Condition	TP, *n*	FP, *n*	FN, *n*	TN, *n*	Analyzed Cases, *n*	Sensitivity, % (95% CI)	Specificity, % (95% CI)	PPV, % (95% CI)	NPV, % (95% CI)	FPR, % (95% CI)
T21	253	32	1	697	983	99.6 (97.8–100.0)	95.6 (93.9–97.0)	88.8 (84.5–92.2)	99.9 (99.2–100.0)	4.4 (3.0–6.1)
T18	62	31	0	890	983	100.0 (94.2–100.0)	96.6 (95.3–97.7)	66.7 (56.1–76.1)	100.0 (99.6–100.0)	3.4 (2.3–4.7)
T13	6	22	0	955	983	100.0 (54.1–100.0)	97.7 (96.6–98.6)	21.4 (8.3–41.0)	100.0 (99.6–100.0)	2.3 (1.4–3.4)
SCAs	85	145	4	749	983	95.5 (88.9–98.8)	83.8 (81.2–86.1)	37.0 (30.7–43.5)	99.5 (98.6–99.9)	16.2 (13.9–18.8)

The performance estimates were derived from a clinically selected NIPT–karyotyping subgroup referred for amniocentesis and should not be interpreted as population-based NIPT screening estimates. Because PPV and NPV depend on the prevalence of the target condition and the clinical composition of the tested population, these values should be interpreted within this referral setting and should not be generalized to unselected prenatal screening populations. The analyses included 983 evaluable cases after excluding 316 inconclusive NIPT results and one case with a no-growth karyotype result. **Abbreviations:** CI, confidence interval; FN, false negative; FP, false positive; FPR, false-positive rate; NIPT, non-invasive prenatal testing; NPV, negative predictive value; PPV, positive predictive value; SCAs, sex chromosome abnormalities; T13, trisomy 13; T18, trisomy 18; T21, trisomy 21; TN, true negative; TP, true positive.

**Table 6:** Comparison of karyotype findings with CMA results showing P and LP.

CMA Finding	CNV Type	CNV Size	Clinical Significance	Karyotype Finding	*n*
arr 1p12p11.2(119,535,334_121,315,484)x3, arr Xp22.31(6,486,490_8,156,174)x1	Gain; Loss	1.8 Mb; 1.7 Mb	LB; P	47,U,+mar	1
arr 2p25.3q37.3(12,770_242,783,384)x2~3	Gain	242.8 Mb	P	46,U	1
arr 2q13(110,458,409_111,388,468)x3, 10q26.13q26.3(127,329,506_135,427,143)x1	Gain; Loss	930 kb; 8.1 Mb	VUS; LP	46,U	1
arr 3p26.3p26.1(61,892_4,190,422)x1, arr 3p26.1(5,975,580_7,431,832)x1, arr 7q31.31q36.3(117,822,870_159,119,707)x3	Loss; Loss; Gain	4.1 Mb; 1.5 Mb; 41.3 Mb	VUS; LB; P	46,U,der(3)t(3;7)(p25;q31.3)	1
arr 4p16.3p11(68,346_49,054,680)x3	Gain	49.0 Mb	P	46,U,+4,der(4;14)(p10;q10)	1
arr 6q27(165,912,970_170,919,482)x1, arr 11p14.3(25,209,939_25,706,990)x1	Loss; Loss	5.0 Mb; 497 kb	P; B	46,U	1
arr 7q11.23(72,739,729_74,257,046)x1, arr 13q31.3q32.1(94,162,371_95,205,884)x3	Loss; Gain	1.5 Mb; 1.0 Mb	P; LB	46,U	1
arr 7q36.1(149,921,667_152,081,462)x1	Loss	2.2 Mb	LP	46,U	1
arr (8)x2 hmz, arr 15q11.2(22,770,422_23,276,605)x1	LOH; Loss	-; 506 kb	VUS; P	46,U	1
arr 8p23.2(3,728,020_5,938,496)x3, arr (21)x3	Gain; Gain	2.2 Mb; -	LB; P	47,U,+21	1
arr (9)x2~3	Gain	-	P	mos 47,U,+9[24]/47,U,inv(7)(q11.23q21.2),+9[3]/47,U,+12[3]/46,U[9]	1
arr 11p11.12(51,127,108_51,581,438)x4, arr 15q11.2(22,770,422_23,276,605)x1	Gain; Loss	454 kb; 506 kb	LB; P	46,U	1
arr 11p15.5(230,616_678,561)x1, arr 11p15.5p15.4(692,384_5,072,822)x3, arr 18p11.32p11.31(136,227_5,975,760)x3	Loss; Gain; Gain	447 kb; 4.4 Mb; 5.8 Mb	VUS; P; P	46,U,add(11)(p15.5)	1
arr 11q23.3q25(116,711,974_134,938,470)x3, arr 22q11.1q11.21(16,888,900_20,295,420)x3	Gain; Gain	18.2 Mb; 3.4 Mb	P; P	47,U,+mar	1
arr 11q24.1q25(122,733,817_131,997,730)x1	Loss	9.3 Mb	P	46,U,der(3)t(3;11)(q25.3;q23.3)del(11)(q24.1q25),der(11)t(3;11)	1
arr (12)x2~3	Mosaic trisomy 12	-	P	mos 47,U,+12[13]/46,U[7]	1
arr 13q31.1q34(85,186,507_115,107,733)x1~2	Loss	29.9 Mb	P	46,U	1
arr (15)x2~3	Gain	-	P	mos 47,U,+15[4]/46,U[16]	1
arr 15q25.1q26.3(80,918,745_102,429,112)x3, arr 18q23(75,310,478_78,014,123)x1	Gain; Loss	21.5 Mb; 2.7 Mb	P; VUS	46,U,add(18)(q23)	1
arr 15q11.2q13.1(22,770,422_29,239,991)x1	Loss	6.5 Mb	P	46,U,del(15)(q11.2q13)	1
arr 16p13.11p12.3(15,359,140_18,198,455)x1	Loss	2.8 Mb	P	46,U	1
arr 17p12(13,980,350_15,412,809)x3	Gain	1.4 Mb	P	46,U	1
arr 17q12(34,831,963_36,350,005)x3	Gain	1.5 Mb	P	46,U	1
arr (18)x3	Gain	-	P	47,U,+18	2
arr 19q13.32q13.43(47,689,921_58,956,888)x3	Gain	11.3 Mb	P	46,U,der(8)t(8;19)(p23.3;q13.3)	1
arr (21)x3	Gain	-	P	47,U,+21	9
arr 21q11.2q22.3(15,006,457_48,097,372)x3	Gain	33.1 Mb	P	47,U,+21	2
arr (22)x2~3	Gain	-	P	46,U	1
arr (22)x2~3	Mosaic trisomy 22	-	P	mos 47,U,+22[2]/46,U[13]	1
arr 22q11.21(18,648,855_21,800,471)x1	Loss	3.1 Mb	P	46,U	1
arr 22q11.21(18,917,030_21,465,662)x1	Loss	2.5 Mb	P	46,U	1
arr 22q11.21(18,917,031_21,804,886)x3	Gain	2.9 Mb	P	46,U	1
arr (X)x1~2	Loss	-	P	mos 45,X[3]/46,XX[12]	1
arr (X)x1~2	Loss	-	P	mos 45,X[16]/46,XY[4]	1
arr (Xp)x1	Loss	-	P	46,X,del(X)(p10)	1
arr Xp22.31(6,486,490_8,156,174)x1	Loss	1.7 Mb	P	46,U	1
arr Xp22.31(6,486,490_8,156,174)x0, arr Yp11.2(7,133,574_7,849,211)x0	Loss; Loss	1.7 Mb; 715 kb	P; LB	46,U	1
arr(X)x2,(Y)x1,(1–22)x3	Gain	-	P	69,XXY	1
Total					48 cases

Rows with multiple CMA findings may include VUS, LB, or B findings when at least one P/LP CNV was identified. U denotes an undisclosed sex chromosome complement, in accordance with ISCN 2024. **Abbreviations:** B, benign; CMA, chromosomal microarray analysis; CNV, copy number variant; hmz, homozygosity; kb, kilobase; LB, likely benign; LOH, loss of heterozygosity; LP, likely pathogenic; Mb, megabase; P, pathogenic; VUS, variant of uncertain significance.

**Table 7:** Comparison of karyotype findings with CMA results showing VUS.

CMA Finding	Finding Type	Size	Clinical Significance	Karyotype Finding
arr (1)x2 hmz	Homozygosity	-	VUS	mos 47,U,+mar[12]/46,U[40]
arr 1p31.1(80,220,589_80,734,553)x3, arr 2q36.1q36.3(224,484,001_227,889,559)x3	Gain; Gain	514 kb; 3.4 Mb	B; VUS	46,U
arr 2p16.3p16.1(52,654,662_59,434,775)x3	Gain	6.8 Mb	VUS	46,U,dup(2)(p16.3p16.1)
arr 2q36.1q36.2(225,104,028_225,341,235)x1	Loss	237 kb	VUS	46,U
arr 6q21q22.1(107,221,059_116,165,572)x3	Gain	8.9 Mb	VUS	46,U
arr 15q21.1q22.2(48,681,302_60,151,916)x2 hmz	Homozygosity	11.5 Mb	VUS	46,U
arr Yp11.31q11.221(2,650,141_16,158,655)x2, arr Yq11.221q11.23(16,184,175_28,799,937)x0	Gain; Loss	13.5 Mb; 12.6 Mb	VUS; VUS	mos 46,X,+mar[5]/46,XY[15]
arr Yq11.221q12(16,591,381_59,363,566)x0, arr Yp11.31q11.221(10,001_16,591,381)x2	Loss; Gain	42.8 Mb; 16.6 Mb	LB; VUS	46,X,+mar
Total				8 cases

Rows with multiple CMA findings may include benign findings when at least one VUS was identified. U denotes an undisclosed sex chromosome complement, in accordance with ISCN 2024. **Abbreviations:** B, benign; CMA, chromosomal microarray analysis; hmz, homozygosity; kb, kilobase; Mb, megabase; VUS, variant of uncertain significance.

**Table 8:** Comprehensive comparison of NIPT, CMA, and karyotyping results across 78 high-risk cases.

NIPT Results	CMA Finding	Finding Type	Size	Clinical Significance	Karyotype Finding	*n*
**Low-risk**						**23**
Low-risk	arr(1–23)x2	-	-	-	46,U	19
Low-risk	arr(1–23)x2	-	-	-	mos 92,XXXX[9]/46,XX[11]	1
Low-risk	arr 6p11.2p11.1(58,142,042_58,759,015)x4	Gain	617 kb	LB	46,U	1
Low-risk	arr 8p23.2(3,728,020_5,938,496)x3	Gain	2.2 Mb	LB	46,U	1
Low-risk	arr 15q21.1q22.2(48,681,302_60,151,916)x2 hmz	LOH	11.5 Mb	VUS	46,U	1
**High-risk**						**30**
T9 high-risk	arr(1–23)x2	-	-	-	46,U	1
T7 high-risk	arr(1–23)x2	-	-	-	46,U	13
T3 high-risk	arr(1–23)x2	-	-	-	46,U	1
T21 high-risk	arr(1–23)x2	-	-	-	46,U	1
T21 high-risk	arr (21)x3	Gain	-	P	47,U,+21	3
T18 high-risk	arr(1–23)x2	-	-	-	46,U	1
T15 high-risk	arr(1–23)x2	-	-	-	46,U	4
Sex chromosome abnormality high-risk	arr(1–23)x2	-	-	-	46,U	1
Sex chromosome abnormality high-risk	arr Yq11.221q12(16,591,381_59,363,566)x0, arr Yp11.31q11.221(10,001_16,591,381)x2	Loss; Gain	42.8 Mb; 16.6 Mb	LB; VUS	46,X,+mar	1
Microduplication	arr(1–23)x2	-	-	-	46,U	1
Microduplication	arr 3p26.3p26.1(61,892_4,190,422)x1, arr 3p26.1(5,975,580_7,431,832)x1, arr 7q31.31q36.3(117,822,870_159,119,707)x3	Loss; Loss; Gain	4.1 Mb; 1.5 Mb; 41.3 Mb	VUS; LB; P	46,U,der(3)t(3;7)(p25;q31.3)	1
Microdeletion	arr(1–23)x2	-	-	-	46,U	2
**Inconclusive**						**25**
Inconclusive	arr(1–23)x2	-	-	-	46,U	18
Inconclusive	arr 3q26.32q26.33(178,841,988_179,431,962)x3	Gain	590 kb	LB	46,U	1
Inconclusive	arr 6q21q22.1(107,221,059_116,165,572)x3	Gain	8.9 Mb	VUS	46,U	1
Inconclusive	arr 11p11.12(51,127,108_51,581,438)x4, arr 15q11.2(22,770,422_23,276,605)x1	Gain, Loss	454 kb, 506 kb	LB, P	46,U	1
Inconclusive	arr 15q25.1q26.3(80,918,745_102,429,112)x3, arr 18q23(75,310,478_78,014,123)x1	Gain; Loss	21.5 Mb; 2.7 Mb	P; VUS	46,U,add(18)(q23)	1
Inconclusive	arr 17p12(13,980,350_15,412,809)x3	Gain	1.4 Mb	P	46,U	1
Inconclusive	arr (18)x3	Gain	-	P	47,U,+18	1
Inconclusive	arr Yp11.31q11.221(2,650,141_16,158,655)x2, arr Yq11.221q11.23(16,184,175_28,799,937)x0	Gain; Loss	13.5 Mb; 12.6 Mb	VUS; VUS	mos 46,X,+mar[5]/46,XY[15]	1
**Total**						**78**

**Abbreviations:** CMA, chromosomal microarray analysis; hmz, homozygosity; kb, kilobase; LB, likely benign; LOH, loss of heterozygosity; Mb, megabase; NIPT, non-invasive prenatal testing; P, pathogenic; VUS, variant of uncertain significance.

## Data Availability

The data presented in this study are not publicly available due to privacy, ethical, and institutional restrictions related to clinical prenatal diagnostic data. The data may be available from the corresponding author upon reasonable request and subject to approval by the relevant institutional review board and data-holding institution.
